# Layer by Layer Assembled Chitosan-Coated Gold Nanoparticles for Enhanced siRNA Delivery and Silencing

**DOI:** 10.3390/ijms22020831

**Published:** 2021-01-15

**Authors:** Elnaz Shaabani, Maryam Sharifiaghdam, Herlinde De Keersmaecker, Riet De Rycke, Stefaan De Smedt, Reza Faridi-Majidi, Kevin Braeckmans, Juan C. Fraire

**Affiliations:** 1 Department of Medical Nanotechnology, School of Advanced Technologies in Medicine, Tehran University of Medical Sciences, Tehran, Iran; elnaz.shaabanisichani@ugent.be (E.S.); maryam.sharifiaghdam@ugent.be (M.S.); 2 Laboratory of General Biochemistry and Physical Pharmacy, Faculty of Pharmacy, Ghent University, B-9000 Ghent, Belgium; herlinde.dekeersmaecker@ugent.be (H.D.K.); stefaan.desmedt@ugent.be (S.D.S.); juan.fraire@ugent.be (J.C.F.); 3 Center for Advanced Light Microscopy, Ghent University, 9000 Ghent, Belgium; 4 Department of Biomedical Molecular Biology, Ghent University, 9052 Ghent, Belgium and VIB Center for Inflammation Research, 9052 Ghent, Belgium; riet.derycke@ugent.be; 5 Ghent University Expertise Centre for Transmission Electron Microscopy and VIB BioImaging Core, 9052 Ghent, Belgium

**Keywords:** gold nanoparticles, Chitosan, layer by layer assembly, siRNA, gene delivery, endosomal escape

## Abstract

Delivery of small interfering RNA (siRNA) provides one of the most powerful strategies for downregulation of therapeutic targets. Despite the widely explored capabilities of this strategy, intracellular delivery is hindered by a lack of carriers that have high stability, low toxicity and high transfection efficiency. Here we propose a layer by layer (LBL) self-assembly method to fabricate chitosan-coated gold nanoparticles (CS-AuNPs) as a more stable and efficient siRNA delivery system. Direct reduction of HAuCl_4_ in the presence of chitosan led to the formation of positively charged CS-AuNPs, which were subsequently modified with a layer of siRNA cargo molecules and a final chitosan layer to protect the siRNA and to have a net positive charge for good interaction with cells. Cytotoxicity, uptake, and downregulation of enhanced Green Fluorescent Protein (eGFP) in H1299-eGFP lung epithelial cells indicated that LBL-CS-AuNPs provided excellent protection of siRNA against enzymatic degradation, ensured good uptake in cells by endocytosis, facilitated endosomal escape of siRNA, and improved the overall silencing effect in comparison with commercial transfection reagents Lipofectamine and jetPEI^®^. Therefore, this work shows that LBL assembled CS-AuNPs are promising nanocarriers for enhanced intracellular siRNA delivery and silencing.

## 1. Introduction

Small interfering RNA (siRNA) is a powerful therapeutic technology which induces post-transcriptional gene silencing via translation inhibition or by cleavage of the target mRNA by incorporating into the RNA-induced silencing complex (RISC) in the cytoplasm [1,2]. Fast enzymatic digestion, limited cellular uptake, inefficient release from endosomes, and lack of cell-specific targeting are the major obstacles of using naked (free) siRNA [3,4,5]. Thus, the therapeutic application of siRNA molecules requires suitable carriers to allow them to be delivered inside target cells in a safe and effective manner. Many different delivery systems have been explored to date. These include viral [5] and non-viral vectors [6,7], each of which come with their own set of advantages and disadvantages. For example, although viral vectors are highly efficient, they are associated with safety concerns such as inherent immunogenicity [8], mutagenesis [9], oncogenic potential, and inflammation [10]. These concerns have triggered the development of non-viral vectors for siRNA delivery including cationic lipids, polymers, peptides and inorganic nanoparticles [7,11,12]. While non-viral vectors are considered to be safer with a reduced immunogenic response, they come with their own drawbacks. For instance, cationic liposomes can suffer from low colloidal stability, difficulties in controlled release, fast elimination in the body, and poor targeting [13]. Polypeptides for gene delivery have limited efficiency and are associated with toxic side effects [14]. In addition, cationic polymer-based carriers can suffer from low stability [15] as well as poor cell recognition and internalization [16]. In spite of those challenges, non-viral vectors remain of great current interest given their better safety profiles and ease of production at an affordable costs [17].

One of the most widely used polymers for the design of nanocarriers for siRNA delivery is chitosan [18,19,20]. Chitosan is a linear biopolymer consisting of randomly repeating D-glucosamine and N-acetyl-d-glucosamine units [21]. Chitosan is a weak polybase with a pKa around 6.5 which offers the advantage of being biodegradable and biocompatible while at the same time being highly positively charged at a pH below the pKa so that it can easily form electrostatic complexes with nucleic acids [22]. On the downside, it suffers from poor stability [23,24] and an undefined structural composition [25]. While improvements have been suggested by making blends with other polymers or modifying its chemical structure [23,26], there is still a need for increasing the stability of chitosan as a gene delivery vehicle. Inorganic nanoparticles like gold nanoparticles (AuNPs) have attracted great interest because of several advantages including their simple synthesis, tunable size and surface properties, good biocompatibility, and multifunctional capabilities [27,28,29]. These unique properties make AuNPs attractive stabilizing scaffolds for gene delivery vehicles. In particular, AuNPs can be used as scaffolds for layer-by-layer (LBL) self-assembly, which is a widely used technique to deposit multiple layers of positively and negatively charged polymers onto surfaces of films or nanoparticles [30,31,32]. Such hybrids of cationic polymers and inorganic nanoparticles combine the advantages of both systems to achieve increased gene delivery efficacy [33]. For instance, LBL assembled AuNP-siRNA have been prepared with polymers that can induce endosomal escape, such as polyethylenimine (PEI) [34,35,36,37,38]. However, release of siRNA in the cellular cytoplasm remains limited due to the high binding affinity between AuNPs-PEI and siRNA [39]. In addition, PEI is often associated with high cytotoxicity due to inducing membrane perturbations and chromosome aberrations [40].

Therefore, in this study we propose LBL assembled AuNP-siRNA in combination with chitosan as a biocompatible and biodegradable cationic polymer to obtain highly stable gene delivery carriers for efficient intracellular siRNA delivery. AuNPs capped with chitosan (CS-AuNPs) are synthesized by using chitosan as both the reducing and stabilizing agent [41,42]. Next, negatively charged siRNA is incorporated as the next layer on top of the positively charged CS-AuNPs. Finally, a third chitosan layer is applied to protect the siRNA and endow the particles with a net positive charge which allows them to easily adsorb to the negatively charged cell membrane, hence facilitating endocytic uptake.

**Scheme 1:**
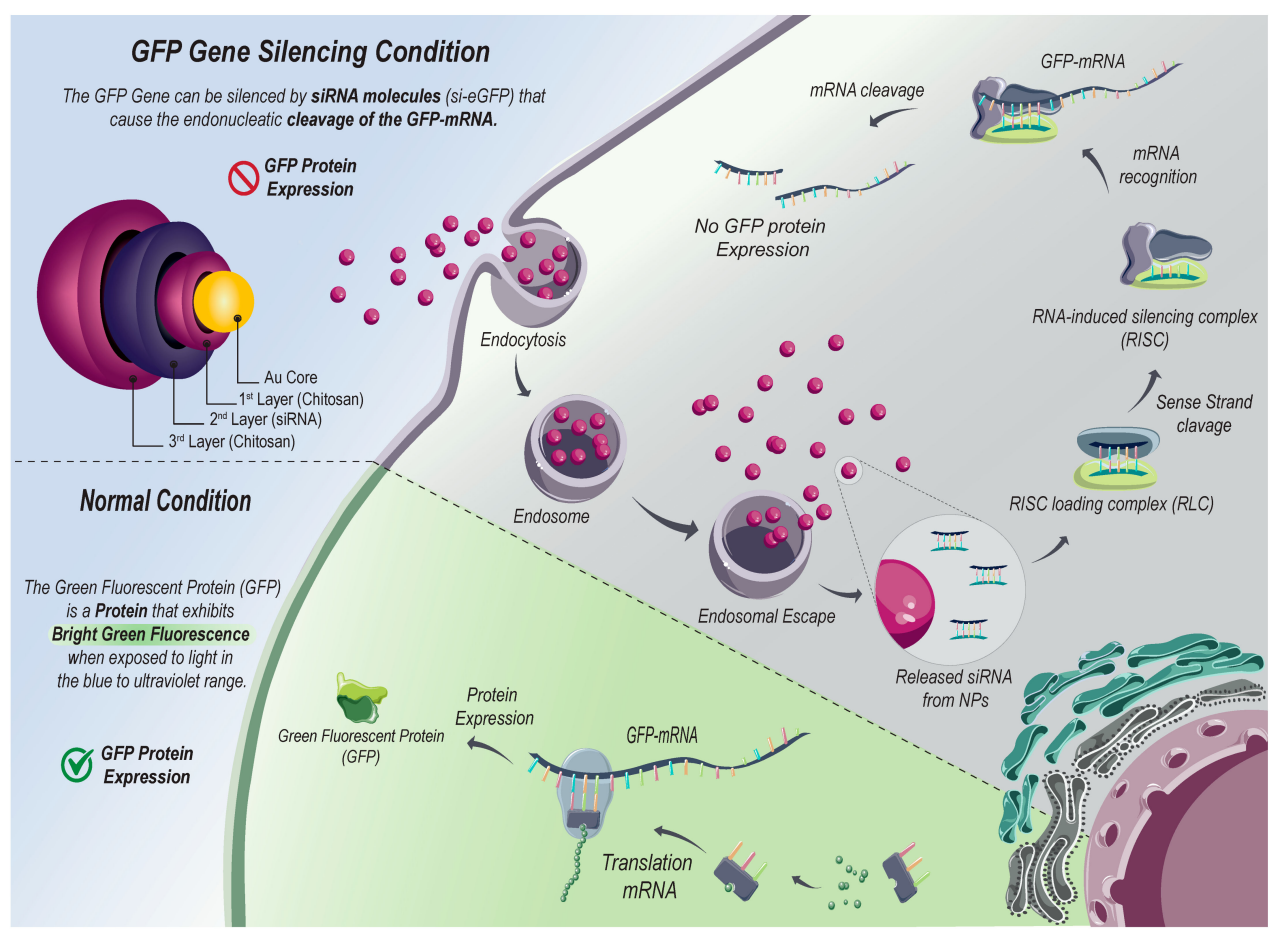
**Schematic representation of LBL-CS-AuNPs for siRNA delivery.** Under normal conditions, H1299-eGFP cells continuously express enhanced Green Fluorescent Protein (eGFP). When LBL-CS-AuNPs are taken up by cells via endocytosis, chitosan can induce endosomal escape. Then, the released siRNA (si-eGFP) is processed by the RNA-induced silencing complex (RISC), which targets and cleaves GFP-mRNA. The cleavage of GFP-mRNA leads to decreased GFP expression and a diminished green fluorescence intensity of H1299-eGFP cells.

Long term stability in storage buffer, stability in different media, and siRNA release are investigated. siRNA gene silencing is subsequently evaluated in H1299 cells stably expressing green fluorescent protein (H1299-eGFP) (as shown in Scheme 1) as a model that allows for an easy readout of siRNA silencing. Cellular uptake, endosomal escape transfection efficiency and cell viability are all studied. We find that the here presented AuNP-based carriers show better stability and efficacy than commercial transfection agents (Lipofectamine (cationic lipid mediated transfection) and jetPEI^®^ (cationic polymer)) or nanocarriers prepared from chitosan alone (CSNPs).

## 2. Results

### 2.1. Preparation and Characterization of Chitosan-Capped AuNPs (CS-AuNPs)

**Figure 1:**
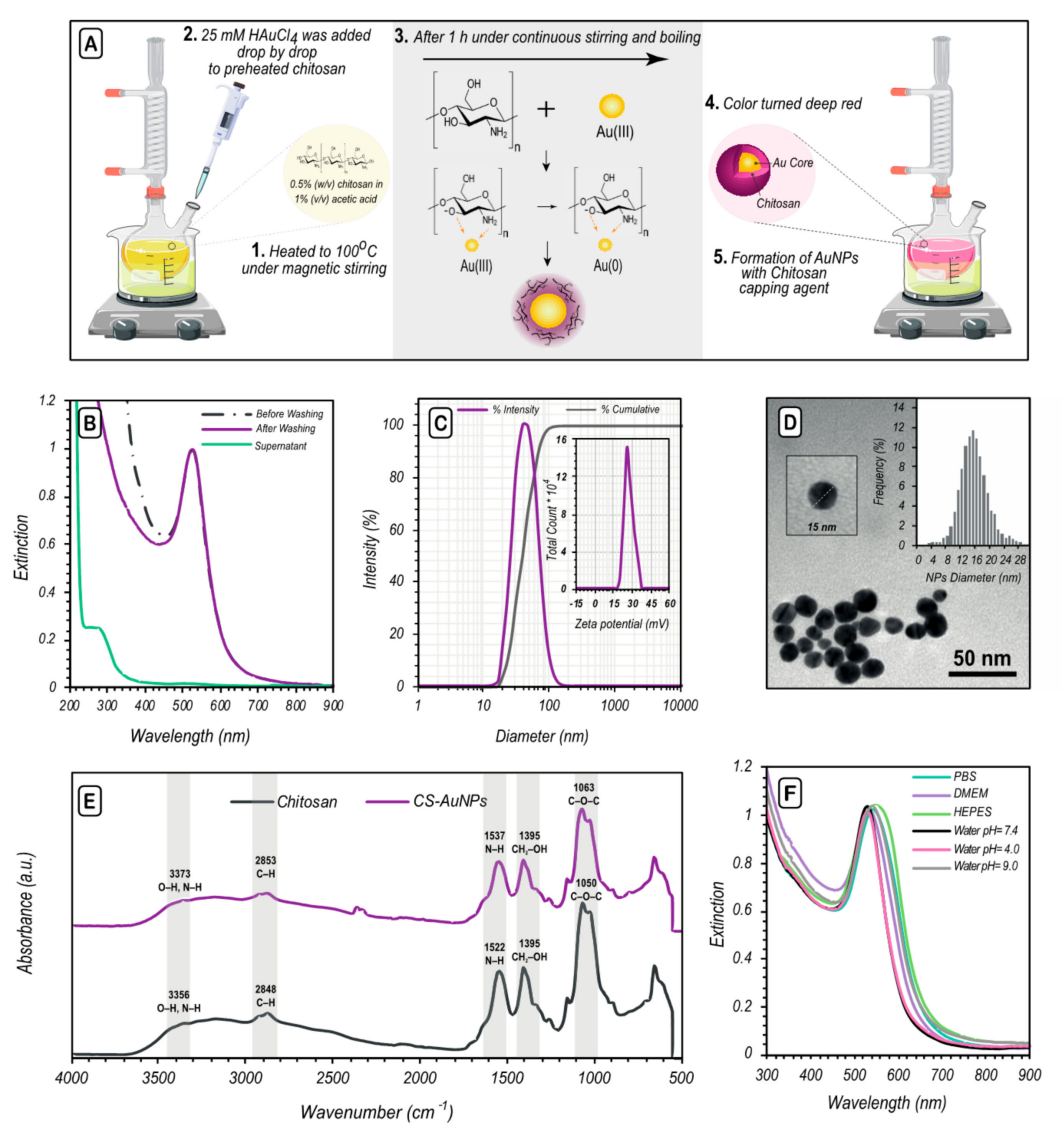
**Characterization of Chitosan-capped AuNPs (CS-AuNPs),** (**A**) Schematic representation of the synthesis of AuNPs using chitosan as a capping and reducing agent. (**B**) UV-Vis spectra of CS-AuNPs before and after washing of unreacted chitosan by centrifugation. (**C**) Representative hydrodynamic size distribution of CS-AuNPs, measured by DLS. The grey curve is the corresponding cumulative distribution. The inset shows the zeta potential distribution. (**D**) TEM images of CS-AuNPs confirming the formation of spherical nanoparticles, which were faceted (inset). The size distribution from measuring >800 nanoparticles revealed an average particle core size of 15.0 ± 0.5 nm. (**E**) FTIR spectra of Chitosan and CS-AuNPs, confirming successful capping of the NPs by Chitosan. (**F**) UV-Vis spectra of CS-AuNPs in different media and pH conditions as indicated in the legend.

By direct reduction of aqueous HAuCl_4_ solution with chitosan (Figure 1A), spherical gold nanoparticles were synthesized as confirmed by the appearance of localized surface plasmon resonance (LSPR) at 524 nm in Figure 1B. In order to remove unreacted chitosan from the samples, CS-AuNPs were washed by centrifugation at 22,000× *g*, 4 °C, for 1 h and then dispersed in ddi. water for further use. The position of the LSPR band did not change after the washing step, indicating that the CS-AuNPs did not aggregate during the purification step. The hydrodynamic diameter of CS-AuNPs was measured in ddi. water and showed an average diameter of 40 ± 5 nm with PDI = 0.24 (Figure 1C). As expected, the CS-AuNPs were positively charged and the zeta potential was 26 ± 3 mV (Figure 1C inset), which suggested that chitosan was present on the CS-AuNPs surface as capping agent.

The morphology and size of CS-AuNPs were determined by transmission electron microscopy (TEM), as shown in Figure 1D. The size distribution indicates an average particle core size of 15 ± 1 nm, calculated by image processing for more than 800 particles (Figure 1D inset). TEM images confirmed that the CS-AuNPs have a spherical morphology.

In order to confirm the presence of chitosan on the surface of CS-AuNPs, we characterized the obtained NPs by Fourier-transform infrared (FTIR) spectroscopy as shown in Figure 1E. The characteristic bands of chitosan (3373 cm^−1^ = N-H, O-H stretching band, 2853 cm^−1^ = C-H stretching band, 1537 cm^−1^ = N-H, and 1063 cm^−1^ = C-O stretching band) [43,44] could indeed be observed in the CS-AuNPs infrared spectrum.

For evaluation of the stability of CS-AuNPs, the UV-Visible spectrum of CS-AuNPs was measured in different media (PBS, DMEM and HEPES) and water at different pH values (4.0, 7.4 and 9.0) after 1h incubation. The LSPR position of CS-AuNPs (Figure 1F) only shifted slightly when exposed to the different media and pH conditions. The effect of the refractive index of the different media on the LSPR peak shift was investigated by Mie theory. Figure S1B in the supporting information shows the simulated extinction cross-section spectra of 15 nm AuNPs performed with reported refractive indexes (nr) of PBS (nr = 1.3348) and DMEM (nr = 1.3370) [45]. As can be seen in the figure, the simulations do not depict appreciably changes of the position of the maximum of the LSPR peak with respect of calculations performed in water (nr = 1.33), arguably due to similar refractive index values between the evaluated media. The experimental shifts observed on the LSPR peak are more likely to be linked to complex local changes of the refractive index due to changes on the macromolecular disposition of the polymeric layers as a function of ion concentration and pH. Combined with the absence of spectral features at higher wavelengths due to plasmon coupling effects of clustered NPs, this indicated that there was no aggregation of CS-AuNPs in these conditions and particles remained colloidally stable [46].

### 2.2. siRNA Loading Capability of the CS-AuNPs

Aimed at obtaining stable CS-AuNPs as a siRNA carrier, a layer-by-layer self-assembly approach was used. This implied that CS-AuNPs was first be coated with a layer of siRNA (CS-AuNPs-siRNA) based on electrostatic complexation of negatively charged siRNA to the cationic CS-AuNPs. In a final step, another layer of chitosan was applied (LBL-CS-AuNPs) to protect the siRNA and to obtain a net positive zeta potential for efficient interaction with cells (Scheme 1).

**Figure 2:**
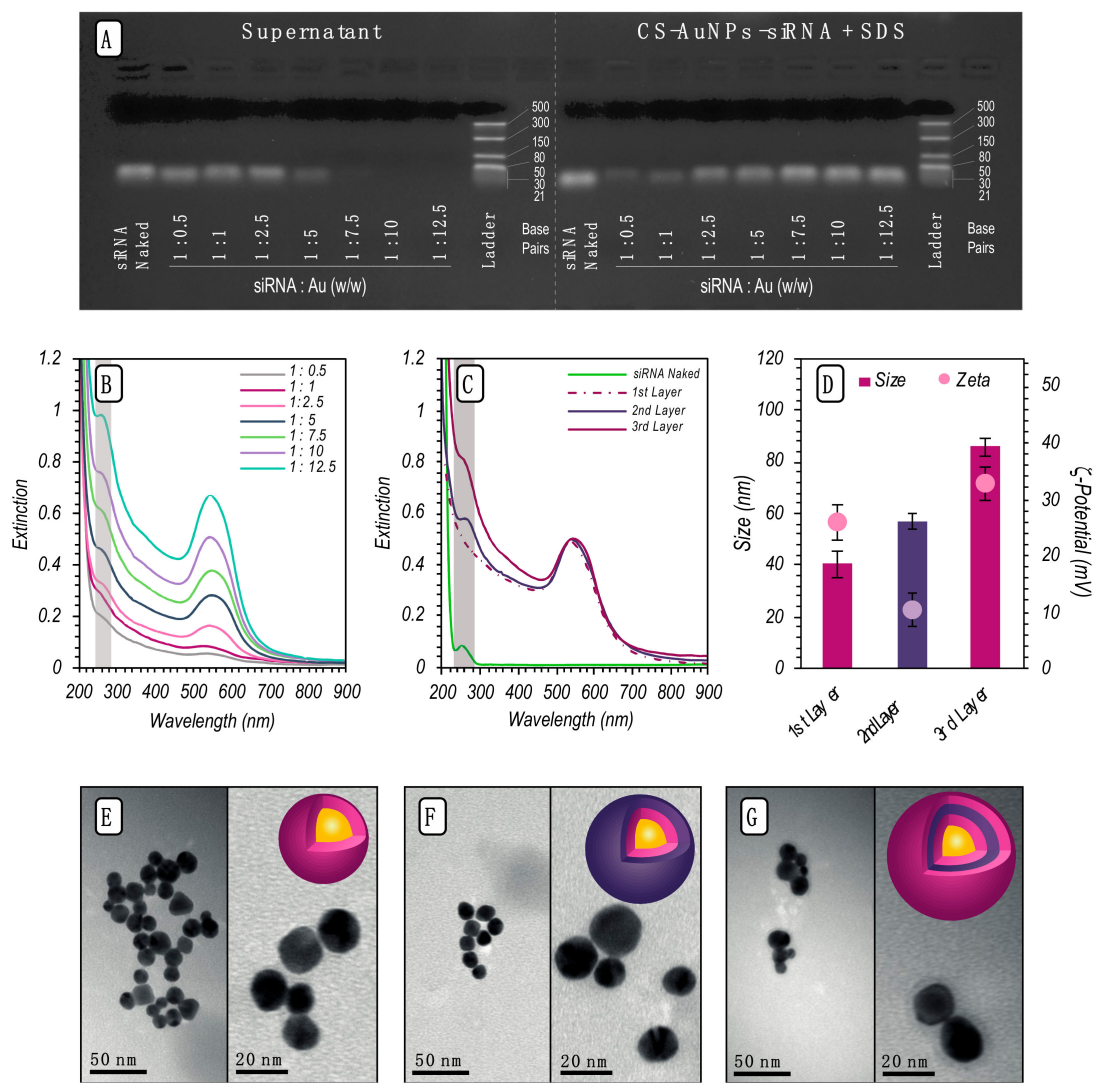
**Synthesis of Layer by Layer (LBL) CS-AuNPs,** (**A**) Agarose gel retardation assay of CS-AuNPs-siRNA complexes for various mass ratios of siRNA to Au ions (siRNA:Au) (1:0.5, 1:1, 1:2.5, 1:5, 1:7.5, 1:10 and 1:12.5). (**B**) UV-Vis spectra of CS-AuNPs-siRNA complexes for various mass ratios. (**C**) UV-Vis spectra upon sequential adsorption of siRNA and chitosan onto CS-AuNPs at weight ratio 1:10. (**D**) Representative particle size and zeta-potential after sequential adsorption of siRNA and chitosan on CS-AuNPs at weight ratio 1:10. (**E**–**G**) TEM images during the three steps of the LBL synthesis process.

The siRNA loading capacity of the CS-AuNPs was evaluated by mixing siRNA and CS-AuNPs at different mass ratios of siRNA to Au, ranging from 1:0.5 to 1:12.5. The corresponding number of siRNA molecules per nanoparticle is shown in Table 1. As shown in Figure 2A (left), as more CS-AuNPs are added to the siRNA, the amount of unbound siRNA in supernatant decreases. In particular, starting from a ratio of 1:7.5 the siRNA band completely disappeared, indicating that all siRNA was successfully complexed to the nanoparticles. Complementary to this, when siRNA was dissociated from the carriers by adding SDS, siRNA bands reappear (Figure 2A right). The signal gradually increases with intensities becoming comparable to naked siRNA starting from a ratio of 1:7.5. These results indicate successful siRNA binding onto the CS-AuNPs by electrostatic interactions, reaching full siRNA loading at ratio 1:7.5. Nevertheless, a weight ratio of 1:10 was selected for further experiments to guarantee working in conditions with maximal siRNA loading.

The process of applying the subsequent layers of siRNA and chitosan on CS-AuNPs was furthermore monitored by UV-visible spectrometry, dynamic light scattering (DLS), zeta potential, and TEM. DNA and RNA bases have a broad absorption band in the UV with maximum absorption around 260 nm. This absorption band was observed in all the UV-Visible spectra recorded from samples after centrifugation (to remove unattached siRNA). The extinction increased gradually from ratio 1:0.5 to 1:12.5, which is due on the one hand to more siRNA being loaded on the CS-AuNPs (at least from ratio 1:0.5 to 1:7.5), combined with gradually more chitosan being present in the sample due to the increasing CS-AuNP concentrations and which also absorbs light in the same wavelength region (Figure 2B). Importantly, the siRNA absorption band remained present after applying the final CS layer, as can be seen in Figure 2C for particles synthetized at ratio 1:10. It is of note that no collective plasmonic modes associated to the optical response of clusters of plasmonic particles were observed at higher wavelengths after deposition of siRNA and chitosan, indicating that LBL-CS-AuNPs did not aggregate and remained stable in colloidal dispersion even 7 days after synthesis (Figure S2).

The LBL process was also monitored for the LBL-CS-AuNP prepared at ratio 1:10 by DLS, revealing a gradual increase in hydrodynamic diameter of the NPs (Figure 2D) from 40 ± 5 to 57 ± 3 and 86 ± 4 nm after applying the second and third layer, respectively. This is also confirmed by a change in zeta potential, starting at 26 ± 3 mV and decreasing to 10 ± 3 mV upon addition of siRNA and increasing again to 33 ± 3 mV with the final chitosan layer (Figure 2D). Figure 2E–G show the TEM images after each coating step. A soft ‘halo’ can be seen around the core particles after applying the third layer, again pointing at successful chitosan coating.

### 2.3. Preparation and Characterization of Chitosan Nanoparticles (CSNPs)

**Figure 3:**
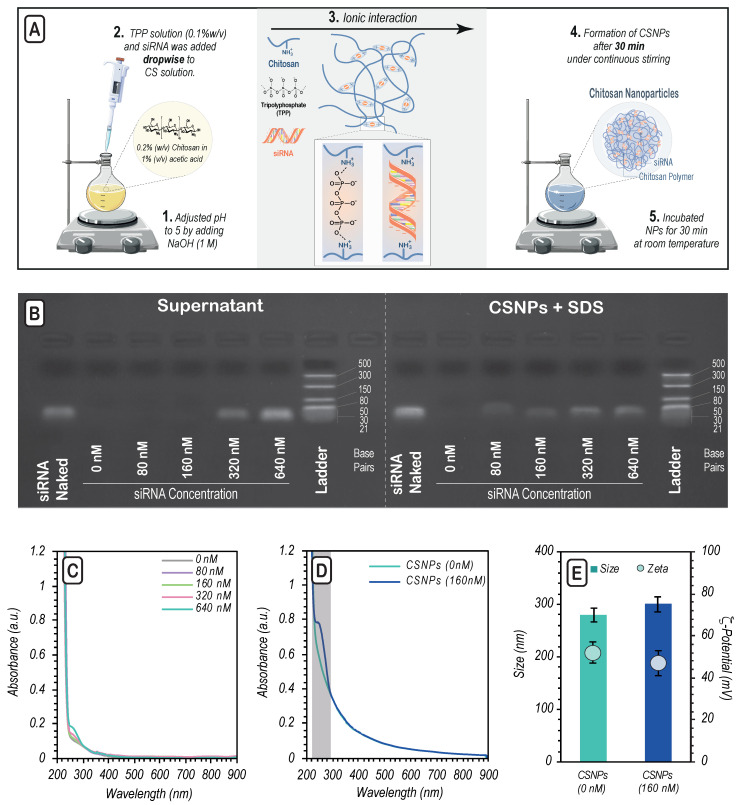
**Synthesis of crosslinked CSNPs for siRNA delivery,** (**A**) Schematic representation of CSNPs synthesis with the crosslinking method. (**B**) Agarose gel retardation assay of CSNPs with siRNA as a function of siRNA concentration. (**C**) UV-Visible spectra of the supernatant obtained after centrifugation, for CSNPs loaded with different amounts of siRNA. (**D**) UV-Visible spectra of CSNPs prepared in the absence or presence of 160 nM siRNA. (**E**) Average hydrodynamic particle diameter and zeta-potential of CSNPs with or without siRNA.

To better judge the added advantage of using AuNPs as a core particle, we additionally synthesized nanocarriers made from chitosan alone (CSNPs). siRNA loaded CSNPs were prepared by mixing different concentrations of siRNA (0–640 nM) with TPP (cross-linker), while keeping the ratio between Tripolyphosphate (TPP) and CS constant, and then adding it dropwise into CS solution under magnetic stirring to form nanoparticles (Figure 3A). As shown in Figure 3B (left), there was no remaining siRNA in the supernatant up until an siRNA concentration of 160 nM, as determined by gel electrophoresis. At higher concentrations unbound siRNA appeared, indicating that the maximum loading capacity was reached. Upon addition of SDS to dissociate siRNA from the CSNPs, fairly identical amounts of siRNA were indeed found for siRNA concentrations of 160 nM, 320 nM, and 640 nM (Figure 3B right). These results showed that starting from an siRNA concentration of 160 nM, maximum loading capacity was achieved. Further confirmation was obtained from the UV-Visible spectrum of the supernatants (Figure 3C), showing the presence of free siRNA only for the two higher concentrations (320 nM and 640 nM), which was not observed for the case of 160 nM. Therefore, from these results, the formulation with 160 nM siRNA was selected for further experiments. The UV-Visible spectrum of these siRNA-CSNPs showed increased absorbance around 260 nm compared to CSNPs (Figure 3D), confirming the presence of siRNA in those nanoparticles. Upon siRNA loading, the nanoparticle’s hydrodynamic diameter slightly increased from 280 ± 13 nm to 300 ± 15 nm, while the zeta potential slightly decreased from +53 ± 5 mV to +47 ± 6 mV (Figure 3E).

### 2.4. Evaluation of siRNA Release and Stability of Nanoparticles

**Figure 4:**
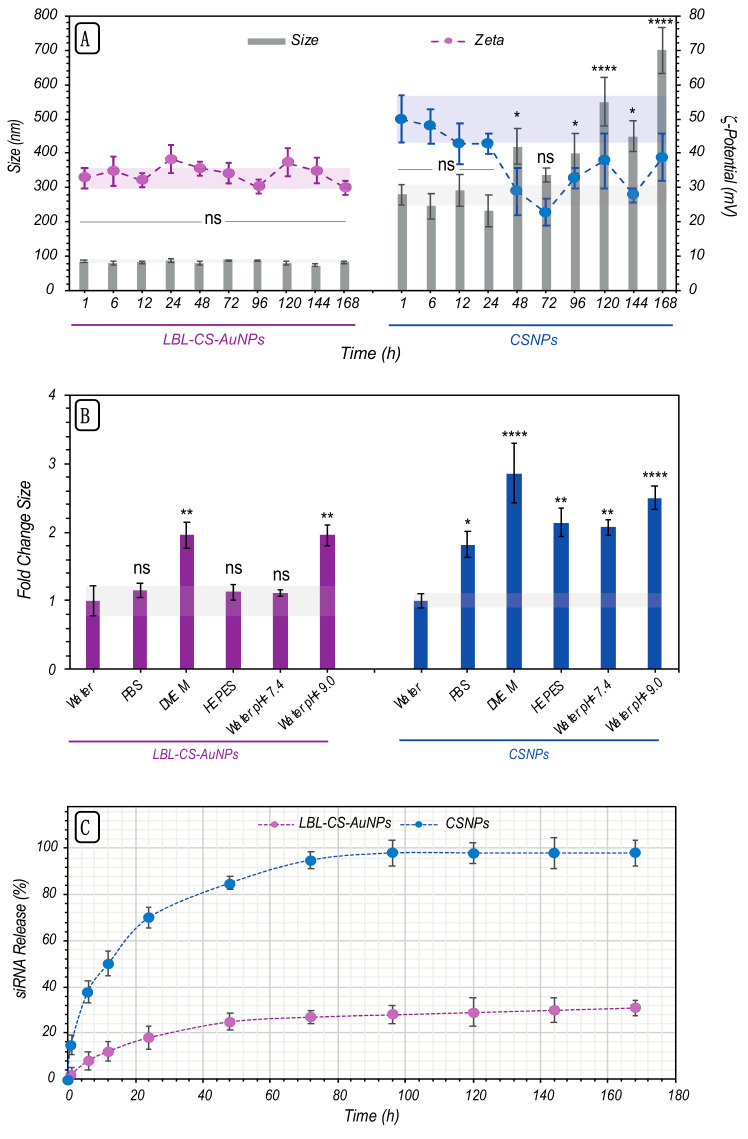
**Comparison of siRNA release and colloidal stability between LBL-CS-AuNPs and CSNPs.** (**A**) Size and zeta potential analysis in DDI water as a function of time for LBL-CS-AuNPs and CSNPs. (**B**) The fold change in particle size of LBL-CS-AuNPs and CSNPs in different media is shown as measured after 1 h incubation. (**C**) Percentage of siRNA released from LBL-CS-AuNPs and CSNPs in pH 7.4 HEPES buffer at 37 °C as a function of time. Data are represented as mean ± the standard error of the mean for a minimum of three independent experiments. Statistical significance, with respect to water, is indicated when appropriate (ns = not significant *p* > 0.05, * *p* ≤ 0.05, ** *p* ≤ 0.01, **** *p* ≤ 0.0001).

One of the main motivations to use a nanocarrier that has a solid core was to improve the stability of the formulations. Evaluation of the size and zeta potential of nanoparticles over time in water (Figure 4A) showed that CSNPs started to aggregate 48 h after synthesis with noticeable changes in size and zeta potential, while LBL-CS-AuNPs remained indeed stable for at least a period of 7 days. When suspended in different media for 1 h, the changes observed for LBL-CS-AuNPs were less drastic, even though an increase in size was observed in water at pH = 9 and in fully supplemented DMEM cell medium (Figure 4B).

Finally, we also monitored siRNA release over time by using UV-Vis from both nanoparticle formulations (Figure 4C). After incubation for 12 h, 24 h, and 7 days, 50%, 70%, and 100% of the siRNA was released from CSNPs, respectively. Instead, the release was only 12%, 18% and 36% for LBL-CS-AuNPs at the same time points. These results show that siRNA release happens more quickly from CSNPs compared to LBL-CS-AuNPs under equal conditions. Together we can conclude that the addition of a gold core and the layer-by-layer design offers clear advantages in terms of both colloidal stability and binding of siRNA.

### 2.5. Cytotoxicity, siRNA Delivery and Gene Silencing Efficiency

**Figure 5:**
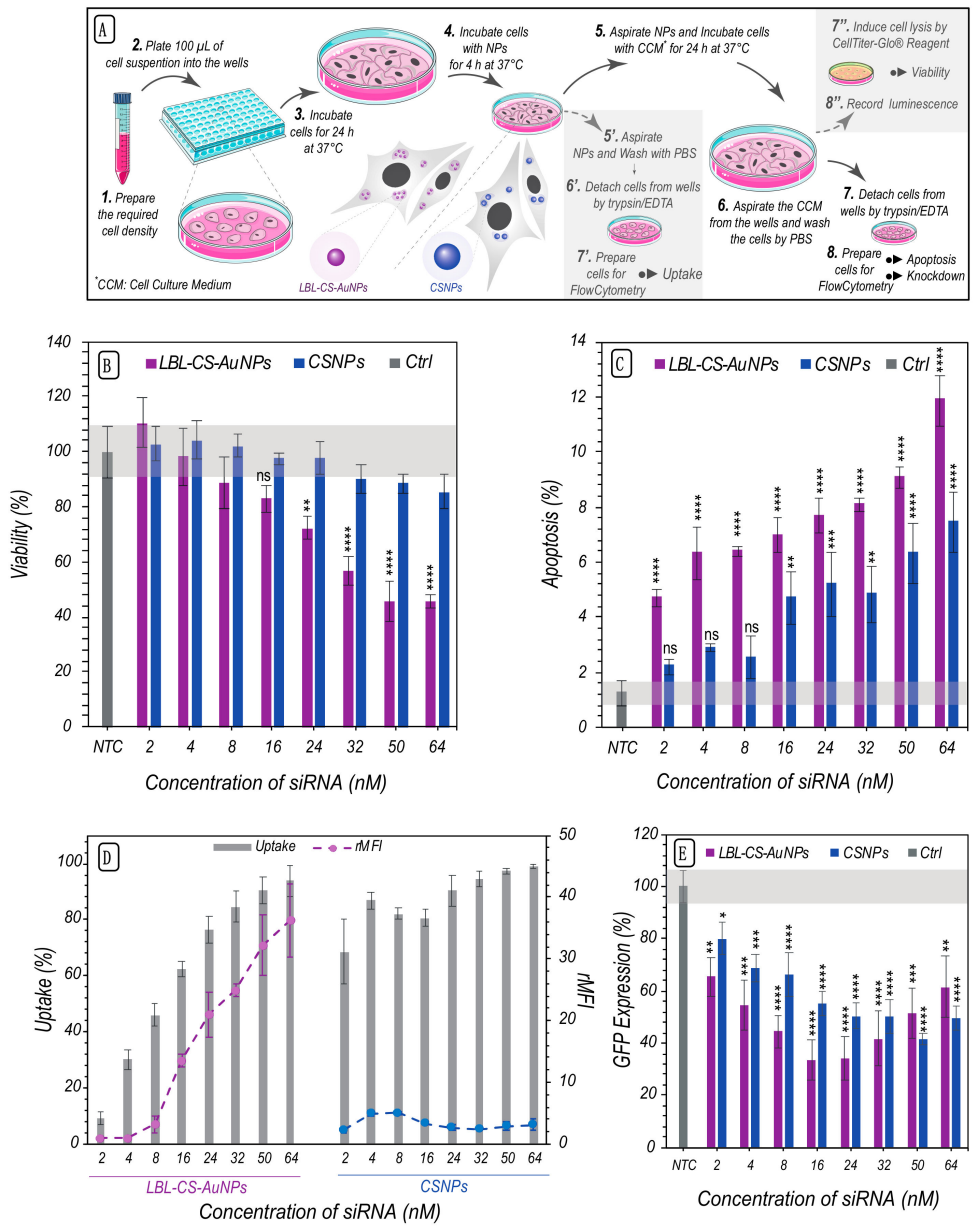
**Comparison of siRNA Delivery, gene silencing efficiency and cytotoxicity between LBL-CS-AuNPs and CSNPs.** (**A**) Schematic representation of the protocol used to evaluation of NPs as siRNA-delivery-carrier. (**B**) Cell viability for different effective concentrations of siRNA was measured by CellTiter-Glo^®^ assay after 4 h incubation with LBL-CS-AuNPs (siRNA:Au 1:10) and CSNPs. (**C**) Apoptosis for different concentrations was assessed based on mitochondrial integrity using DiIC_1_(5) and PI staining. (**D**) Uptake percentage (= % positive cells) and relative mean fluorescence intensities of AF647 siRNA labeled LBL-CS-AuNPs on H1299-eGFP cells were determined by flow cytometry. (**E**) The percent of GFP expression on H1299-eGFP cells was measure based on the mean GFP fluorescence intensity (MFI) of the cell by flow cytometry at different concentrations of LBL-CS-AuNPs loaded with GFP-siRNA. Data are represented as mean ± the standard error of the mean for a minimum of three independent experiments. Statistical significance, with respect to the not treated control (NTC), is indicated when appropriate (ns = not significant *p* > 0.05, * *p* ≤ 0.05, ** *p* ≤ 0.01, *** *p* ≤ 0.001, **** *p* ≤ 0.0001).

The biocompatibility of a carrier is an important consideration for its clinical application. Therefore, we proceeded to evaluate the cytotoxicity profiles of the nanoparticles on H1299-eGFP cells by measuring the cell’s metabolic activity (via CellTiter-Glo^®^ luminescent assay) and the percentage of cells that go into apoptosis (via DiIC_1_(5) and PI staining) after 4 h incubation with NPs followed by 24 h incubation in fresh cell culture medium (Figure 5A). When H1299-eGFP cells were incubated with CSNPs, no toxicity was observed over the whole concentration range studied (Figure 5B). For LBL-CS-AuNPs a constant weight ratio of 1:10 (siRNA:Au) was used. By increasing the nanoparticle concentration added to cells, also the effective siRNA concentration was increased, as summarized in Table 2. The cell viability decreased more quickly for LBL-CS-AuNPs compared to CSNPs for the same effective siRNA concentration. Correspondingly, the percentage of apoptotic cells increased more rapidly for LBL-CS-AuNPs, as can be seen in Figure 5C and Figure S3.

We next proceeded to determine the percentage of uptake of LBL-CS-AuNPs and CSNPs in H1299-eGFP cells. Cell uptake studies were performed using Alexa Flour 647 (AF647) labeled siRNA. Figure 5D shows the percentage of positive cells after 4 h incubation with nanoparticles prepared with the AF647 labeled siRNA.

Flow cytometry data showed that both NPs were taken up by H1299-eGFP cells with the percentage of positive cells gradually increasing as a function of the NP concentration (Figure 5D). At lower concentrations, CSNPs were taken up by more cells, while at the highest concentrations, both NPs were taken up by near 100% of the cells. A striking difference was, however, seen in the relative mean fluorescence intensity (rMFI) per cell, which is related to the number of nanoparticles per cell and which was much higher for LBL-CS-AuNPs, starting from an siRNA concentration of 16 nM.

To evaluate siRNA transfection by the nanoparticles, eGFP-siRNA was used to evaluate downregulation of eGFP in H1299 cells constitutively expressing eGFP. The level of eGFP protein expression was quantified as the mean green fluorescence intensity of the entire cell population via flow cytometry. Results from flow cytometry showed that eGFP expression had indeed decreased 24 h after treatment with the NPs (Figure 5E). At lower concentrations (2 nM to 24 nM of siRNA) LBL-CS-AuNPs showed more eGFP downregulation than CSNP, which is likely due to the fact that more LBL-CS-AuNP are taken up per cell than CSNP. Above 24 nM, the eGFP expression rate increased again for LBL-CS-AuNP, which is likely related to the toxicity observed for such high concentrations of LBL-CS-AuNPs.

**Figure 6:**
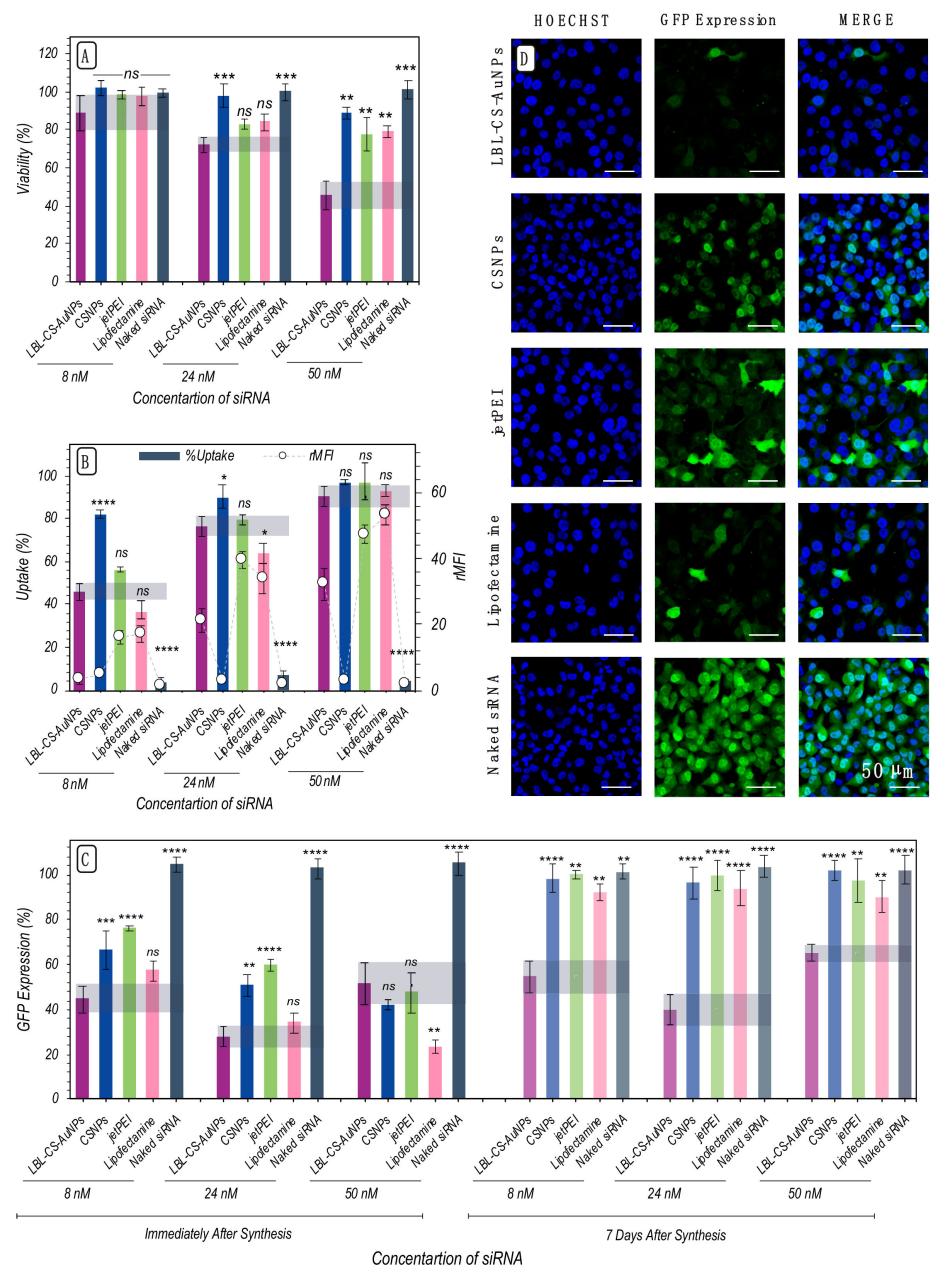
**Evaluating the cytotoxicity, uptake and knockdown efficacy of LBL-CS-AuNPs and CSNPs in comparison with jetPEI^®^ and Lipofectamine as commercial transfection reagents for siRNA**. (**A**) Cell viability was measured by CellTiter GLO assay after 4 h incubation with the different carriers at three effective siRNA concentrations (8 nM, 24 nM, and 50 nM). (**B**) Uptake percentage (= % pos-itive cells) and relative mean fluorescence intensities of AF647 siRNA labeled carriers on H1299-eGFP cells were determined by flow cytometry. (**C**) Percentage of eGFP expression on H1299-eGFP cells treated with the different carriers at three effective siRNA concentrations (8 nM, 24 nM, and 50 nM) for both freshly and 7 days after synthesis. (**D**) Confocal images of H1299-eGFP cells after treatment with different carriers at 24 nM effective concentration of siRNA. The nuclei stained with Hoechst 33,342 are shown in blue and the eGFP expression is shown in green in the fluorescence images. The scale bars correspond to 50 μm. Data are represented as mean ± the standard error of the mean for a minimum of three independent experiments. Statistical signifi-cance, with respect to LBL-CS-AuNPs, is indicated when appropriate (ns = not significant *p* > 0.05, * *p* ≤ 0.05, ** *p* ≤ 0.01, *** *p* ≤ 0.001, **** *p* ≤ 0.0001).

To better appreciate the performance of LBL-CS-AuNPs, they were compared to two popular commercial transfection reagents, Lipofectamine and jetPEI^®^ for three siRNA concentrations (8, 24 and 50 nM). CSNPs were included once more as a reference, next to naked siRNA, which was included as a negative control. The cell viability after treatment with LBL-CS-AuNP, Lipofectamine or jetPEI^®^ was not significantly different at siRNA concentrations of 8 and 24 nM (Figure 6A). At the highest concentration (50 nM), LBL-CS-AuNPs did become more toxic though. Uptake increased with increasing siRNA concentrations for all carriers, as expected (Figure 6B). No significant difference was found between them in terms of the percentage of positive cells for a given siRNA concentration. The amount of uptake per cell (based on rMFI) was, however, systematically higher for the commercial transfection reagents. In terms of eGFP downregulation, LBL-CS-AuNP performed better (8 nM) or equally well (24 nM and 50 nM) as jetPEI^®^ (Figure 6C and Figure S4). This is also visually apparent from the supporting confocal microscopy images in Figure 6D. Compared to Lipofectamine, LBL-CS-AuNP performed similar (8 and 24 nM) or worse (50 nM). The latter result can likely again be attributed to the relatively high extent of cytotoxicity by LBL-CS-AuNP at such high concentrations.

While this confirms efficient cell transfections with LBL-CS-AuNP, their major benefit comes from the added stability over time. Indeed, incorporation of gold nanoparticles as a core was motivated by the aim to improve the long-term stability of the formulations. In line with their previously demonstrated enhanced colloidal stability, their knock-down efficiency 7 days after synthesis remained unaltered, while all other tested carriers and transfection reagents had lost most, if not all, of their activity (Figure 6C). This confirms that the LBL-CS-AuNP design offers superior long-term activity with siRNA downregulation efficiencies that are up to par with the most popular transfection reagents.

### 2.6. Evaluation of Endosomal Escape

Successful endosomal escape and release of siRNA into the cytoplasm are known to be critical prerequisites that a nanocarrier must fulfill for effective siRNA delivery and the consequent knockdown of specific proteins. To investigate the role of endosomal escape in the observed transfection efficiencies, confocal fluorescence microscopy studies were carried out using Alexa Fluor 647-labeled oligonucleotides (AF647 ONs) as cargo for the different nanoparticles and transfection reagents. When the ONs are incorporated into the nanocarrier, their fluorescence is mostly quenched, but after release from the endosomes and dilution into the cytoplasm, their fluorescence enhances again [47,48]. As these ONs accumulate in the nucleus by active transport over time, the presence of a red fluorescent nucleus is indicative of at least one endosomal escape event having happened in a particular cell. By counting on the one hand the total number of nuclei in confocal images (by Hoechst staining) and red fluorescent nuclei on the other hand, the percentage of cells can be calculated in which at least one endosomal escape event has occurred.

**Figure 7:**
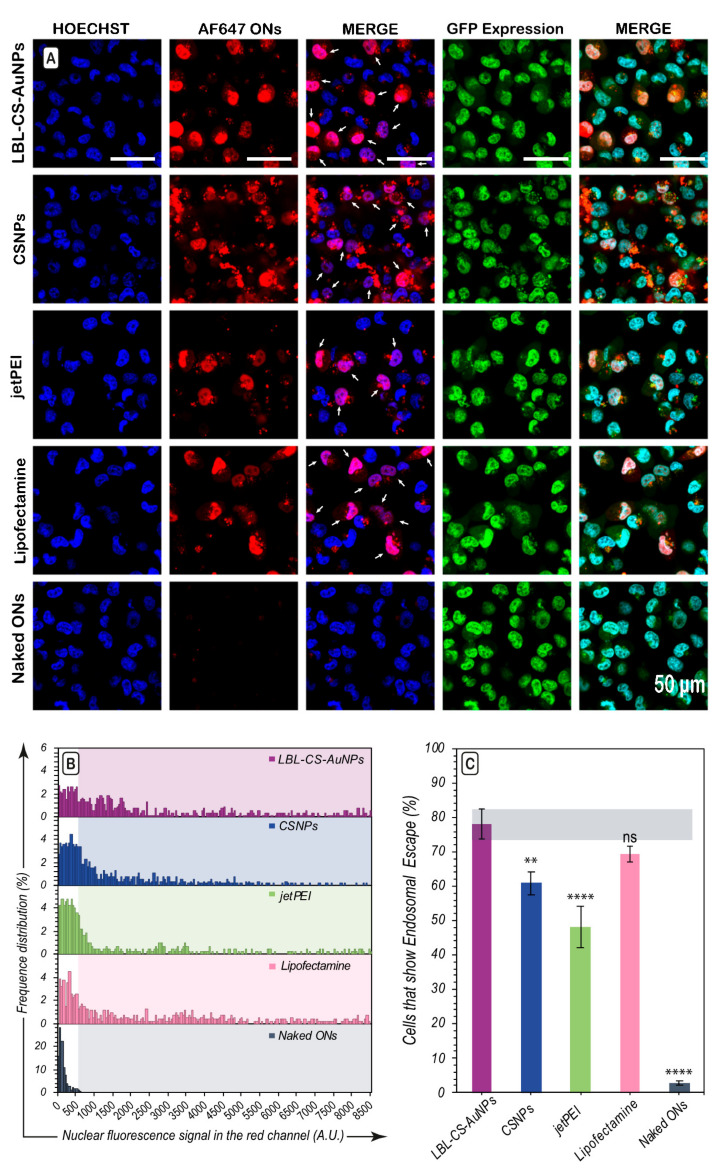
**Evaluating the role of endosomal escape.** (**A**) Representative confocal images 24 h after incubation with AF647-ON loaded carriers in the H1299-eGFP cell line. Hoechst nuclei can be seen in blue, while cells in which endosomal escape occurred show nuclear fluorescence in the red channel due to the release of AF647 ONs from the endosome to the cytoplasm, which finally accumulate in the nuclei (white arrows in 3rd column). The scale bars correspond to 50 μm. (**B**) Histograms of the red fluorescent intensity of cell nuclei for the different nanocarriers. (**C**) The percentage of cells with red nuclei calculated by quantification of at least 500 nuclei. Data are represented as mean ± the standard error of the mean for a minimum of three independent experiments. Statistical significance, with respect to LBL-CS-AuNPs, is indicated when appropriate (ns = not significant *p* > 0.05, ** *p* ≤ 0.01, **** *p* ≤ 0.0001).

Representative microscopy images obtained after 24 h can be seen in Figure 7A for LBL-CS-AuNPs, CSNPs, Lipofectamine, jetPEI^®^, and free AF647 ONs at an effective ON concentration of 24 nM. The presence of red fluorescent nuclei in the microscopy images (Figure 7A) confirmed that endosomal escape took place after incubation with every carrier. Frequency distributions of the nuclear fluorescence signal in the red channel of all H1299 cells per condition, quantified from the confocal images (Figure 7B). The percentage corresponding to the percentage of cells with red nuclei in which escape has happened was calculated by analysis of at least 500 cells from 20 images for each sample, showing endosomal escape in 78%, 60%, 48%, and 69% of the cells for LBL-CS-AuNPs, CSNPs, jetPEI^®^, and Lipofectamine, respectively (Figure 7C). These percentages show that endosomal escape occurred most efficiently for LBL-CS-AuNPs and Lipofectamine-treated cells, in line with the transfection results.

## 3. Discussion

Chitosan is a biocompatible and biodegradable cationic polysaccharide that can form complexes with negatively charged nucleic acids through electrostatic interactions [21,49,50]. The amino groups of chitosan can trigger a “proton sponge effect” [51] which contributes to the endosomal escape of the complex. While PEI does this as well, chitosan is less cytotoxic than PEI, which can interact with proteins leasing ti the induction of apoptosis [52]. In addition, PEI may cause disruptions in the cell membrane, leading to necrotic cell death or even chromosome aberrations [40,53,54].

Despite its advantages, there are hardly any available pharmaceutical products based only on chitosan. This might be a result of the high susceptibility of chitosan to environmental factors (humidity, temperature, and pH) [55], structural instability, and lack of standardized and condensed physical structure. Ongoing research aims at chemically modifying chitosan’s chemical structure to generate more stable formulations. [18,23]. Our study here was performed from a similar point of view. However, rather than chemically modifying chitosan, we explored if the use of a solid gold core may enhance the stability of chitosan-based siRNA carriers while still retaining chitosan’s efficient transfection properties. AuNPs were chosen due to their narrow size distribution, facile synthesis and stable nature [26]. Using a straightforward LBL assembly method, a chitosan-based siRNA carrier was designed.

### 3.1. Nanoparticle Formation, Characterization and Stability

CS-AuNPs have been synthetized in a one-step synthetic method which used chitosan (CS) both as the reducing agent and stabilizer to generate CS-capped AuNPs. The appearance of a LSPR peak at 524 nm in the UV-Visible spectrum and the absence of plasmonic bands associated with agglomeration of nanoparticles confirmed that the CS-AuNPs are stable and do not show aggregation. For the second layer of the LBL coating, different ratios of siRNA were evaluated for siRNA attachment on the surface of the CS-AuNPs. Then, a final CS layer was applied to protect the loaded siRNA from preventing fast release and ensuring efficient uptake by cells and efficient endosomal escape.

Evaluation of siRNA release profiles and colloidal stability indicated that LBL-CS-AuNPs were more stable than CSNPs which are composed of chitosan alone. We hypothesize that the macromolecular organization of the polymer layer on the surface of the gold nanoparticles confers to their high colloidal stability due to the high cationic charge and the steric effect of the chitosan.

### 3.2. Uptake and Transfection Efficiency of Nanoparticles

The biocompatibility of a vector for siRNA delivery is an important consideration. Investigation of metabolic activity and induction of apoptosis showed that CSNPs induced very little toxicity, even at the highest concentration, while for LBL-CS-AuNPs, the toxicity gradually increased with increasing concentration. Chitosan is known to be a biocompatible polymer so the low toxicity by CSNPs is not surprising. The fact that LBL-CS-AuNP induced more toxicity is very likely due to enhanced cellular uptake, as we indeed could observe by using fluorescently labeled siRNA. For CSNPs, the rMFI did not increase substantially with increasing NP concentration, indicating that under the studied concentrations, the uptake machinery was already saturated. Indeed, it has been previously suggested that endocytic uptake pathways may be different for particles of different sizes [56,57]. Therefore, it cannot be excluded that the larger CSNPs are taken up via a different endocytic pathway, which perhaps may saturate more quickly, than the smaller LBL-CS-AuNPs. Regardless of the underlying mechanism, enhanced uptake of LBL-CS-AuNPs resulted in a maximum gene silencing of 76% for LBL-CS-AuNP with 24 nM siRNA, while this remained limited to 49% for CSNPs with 50 nM siRNA.

When comparing transfection efficiencies with two commercial transfection reagents, jet-PEI and Lipofectamine, it was found that CSNPs showed similar effects as jetPEI^®^, while being much less effective than Lipofectamine. LBL-CS-AuNPs at 8 nM, on the other hand, performed even better than Lipofectamine, with similar knockdown efficiencies at higher siRNA concentrations. Importantly, unlike the other carriers (commercial transfection reagents or CSNPs), LBL-CS-AuNPs were still as functional 7 days after synthesis, which is yet another demonstration of superior stability of LBL-CS-AuNPs.

### 3.3. Endosomal Escape Efficiency

In order to explain the experimental observations of the silencing effect, we proceeded to determine and quantify the endosomal escape capacity of all the NPs evaluated. After image analysis (~500 cells for each sample), a direct correlation was found between the extent of endosomal escape and the transfection efficiencies, indicating that the escape from endosomes is one of the main factors in the effectiveness of the siRNA delivery carriers evaluated here.

## 4. Materials and Methods

### 4.1. Materials

HAuCl_4_ and Chitosan (CS, low molecular weight, degree of deacetylation: 80%) were purchased from Sigma-Aldrich (Overijse, Belgium). RPMI-1640, DMEM (without phenol red), L-Glutamine, Penicillin/Streptomycin solution (5000 IU/mL penicillin and 5000 μg/mL streptomycin) (P/S), Fetal Bovine Serum (FBS), Trypan Blue, 0.25% Trypsin-EDTA, and Dulbecco’s phosphate-buffered saline (DPBS) were supplied by Gibco BRL (Merelbeke, Belgium). CellTiter-Glo^®^ Luminescent Cell Viability Assay was purchased from Promega (Leiden, The Netherlands). Hoechst 33,342 was purchased from Molecular Probes (Erembodegem, Belgium). Lipofectamine RNAiMAX reagent was purchased from Invitrogen. jetPEI (jetPRIME^®^) was purchased from Polyplus-transfection^®^ company (Illkirch, France). siRNA against GFP (si-eGFP, sense strand = 5′-CAAGCUGACCCUGAAGUUCtt-3′; antisense strand = 5′-GAACUUCAGGGUCAGCUUGtt-3′) and nontargeted siRNA (si-CTRL, sense strand = 5′-CAAGCUGACCCUGAAGUUCtt-3′; antisense strand = 5′-GAACUUCAGGGUCAGCUUGtt-3′) were purchased from Eurogentec (Seraing, Belgium). For uptake experiments, the si-CTRL duplex was labeled with Alexa Fluor 647 dye at the 5′ end of the sense strand (si-AF647) (Eurogentec, Seraing, Belgium). For endosomal escape AlexaFluor647-labeled oligonucleotides (AF647 ONs) was used (Eurogentec, Seraing, Belgium).

### 4.2. Nanoparticles Synthesis

**Layer by Layer Gold nanoparticles (LBL-CS-AuNPs):**

***Core:*** Gold nanoparticles of 20 nm of diameter capped with chitosan were synthesized by the reduction of HAuCl_4_ directly by chitosan [36,38]. Briefly, 200 mL of 0.5% (*w*/*v*) chitosan solution dissolved in 1% (*v*/*v*) acetic acid was heated to 100 °C under magnetic stirring and reflux. Next, 85 μL of 25 mM HAuCl_4_ was added to preheated chitosan drop by drop and allowed to boil for 1 h under continuous stirring till the color turned deep red indicating the formation of AuNPs. For removing unreacted material, NPs were centrifuged at 22,000 g, 4 °C, for 1 h and dispersed in deionized water for further use.

***Layer by layer assembly of CS-AuNPs:*** To load negatively charged siRNA molecules onto the CS-AuNPs as a second layer, CS-AuNPs were resuspended in 10 mM HEPES buffer with pH = 7 and mixed with siRNA at various weight ratios of siRNA to Au (1:1–12.5) under continuous stirring (400 RPM) incubated for 1 h. Lastly, a final chitosan layer was applied by dispersing the nanoparticles in a 0.5% (*w*/*v*) chitosan solution, which was mixed under continuous stirring for another 1 h (LBL-CS-AuNPs). Excess of chitosan and unattached siRNA was removed by centrifugation at 22,000× *g*, 4 °C, 1 h, and the purified particles were resuspended in RNase-free water.

**Chitosan Nanoparticles (CSNPs):**

CSNPs were synthesized via the ionic gelation method as previously reported [58]. Chitosan (CS) solutions were prepared by dissolving CS in 1% *v/v* acetic acid. The cross-linking agent, Tripolyphosphate (TPP), was prepared by dissolving TPP in deionized water. For siRNA entrapment in CSNPs, siRNA was dissolved in the TPP solution before adding to the CS solution. The pH of CS solution was adjusted to 5 by adding NaOH (1 M) after which TPP solution (0.1% *w*/*v*) with siRNA (0–640 nM) was added dropwise into CS solution (0. 2% *w*/*v*) under magnetic stirring (5:1 weight ratio of chitosan to TPP) at 900 RPM for 30 min to form nanoparticles. The formed nanoparticles were then incubated for another 30 min at room temperature and centrifugated at 15,000 g at 4 °C for 30 min to collect the nanoparticles. The pellets of nanoparticles were resuspended in RNase-free water.

### 4.3. Characterization of Nanoparticles

UV–Visible spectroscopy was used to characterize spectral changes in the localized surface plasmon resonance (LSPR) band of CS-AuNPs in the range of 200–900 nm by using Thermo Scientific NanoDropTM Spectrophotometers. The hydrodynamic size and zeta potential of the particles were measured using a Malvern Zetasizer Nano ZS instrument (Malvern, UK) with a He/Ne laser (633 nm). The morphology and particle size were analyzed by transmission electron microscopy (TEM) (JEOL, Tokyo, Japan) operating at 80 kV at the VIB-UGent. The samples were prepared by depositing a drop (50 µL) of NPs on a formvar/C-coated hexagonal copper grid (EMS G200H-Cu) which was allowed to dry at room temperature and washed 5 times in double-distilled H_2_O. The average particle size was determined of counting about 800 particles using Image J. To confirm the presence of chitosan on CS-AuNPs, their FTIR spectrum was acquired. The washed CS-AuNPs were freeze-dried (Amsco-Finn Aqua GT4 freeze-dryer (GEA, Köln, Germany)) to obtain a dry powder and incorporated into pellets after mixing with KBr powder. Spectra were collected in a BRUKER Vertex 80v Vacuum FT-IR spectrometer (USA) over a range of 4000 cm^−1^ to 400 cm^−1^. 100 µL of CS-AuNPs was dissolved in 1 mL aqua regia (mixture of 36% hydrochloric acid and 65% nitric acid in a 3:1 ratio) after which it was evaporated under heating to reach a volume of 250 µL, after which it was allowed to cool to room temperature and diluted with water to a final volume of 10 mL. Finally, the Au content of those samples was determined by an atomic absorption spectrometer (Varian AA240FS, Varian, Mulgrave, Australia).

### 4.4. Evaluation of siRNA Binding to NPs

For the synthesis of CSNPs, various amounts of siRNA were mixed with TPP and after synthesis, NPs were centrifuged. For evaluation of the siRNA loading capacity of CSNPs, the supernatant was evaluated with gel electrophoresis in order to quantify unbound siRNA. For CS-AuNPs, various weight ratios of CS-AuNPs were mixed with siRNA for 1 h followed by centrifugation at 22,000× *g*, 4 °C, 1 h.

20 µL of the supernatant were mixed with 5 µL of loading buffer and then 20 µL of the mixture was loaded onto 1% agarose gel (UltraPure Agarose, Invitrogen, Erembodegem, Belgium) containing 1:10,000 of Gel-RED^TM^ stain (Biotium, Hayward, CA, USA). To evaluate the siRNA loading capacity of the NPs, 5 μL of a 2% SDS solution was added to 20 µL of NPs to dissociate siRNA from the NPs. The supernatant was prepared in exactly the same way. After loading the samples onto an agarose gel, electrophoresis was carried out in a horizontal gel electrophoresis unit (Bio-Rad Laboratories, Richmond, CA, USA) at 100 V for 30 min in TBE buffer (98 mM Tris, 88 mM Boric acid, 2 mM Na_2_EDTA with pH 8). Then the fluorescence bands were visualized using a Kodak digital science camera (Kodak EDAS 120, Rochester, NY, USA) under UV light (Bio-Rad UV transilluminator 2000, Richmond, CA, USA).

### 4.5. Stability and siRNA Release from the NPs

To evaluate the release profile of siRNA, the separation and analysis method described by Shen et al. was used [59], in which the NPs are directly added into the release medium and sample separation techniques (centrifugation) are used to separate the dispersed nanoparticles from the continuous phase at different time intervals. Briefly, the NP suspension in 10 mM HEPES buffer at pH 7.4 was incubated for 7 days at 37 °C in microtubes equal in number to the time intervals at which measurements will be performed. At different time intervals, one microtube was taken and centrifuged at 22,000× *g* for 1 h at 4 °C. The concentration of siRNA present in the supernatant was measured using a Thermo Scientific NanoDrop^TM^ Spectrophotometers at 260 nm. The hydrodynamic diameter and the zeta potential of NPs diluted in DI water and other media was measured over time by dynamic light scattering (Malvern Zetasizer Nano ZS).

### 4.6. Cell Culture

H1299 cells stably expressing GFP (H1299-eGFP), which are lung epithelial cells derived from metastatic lymph nodes (ATCC-CCL 5803), were used as cell model in this study. In every case, the passage number was kept below 20. H1299-eGFP cells were cultured in Roswell Park Memorial Institute (RPMI) 1640 medium supplemented with 10% FBS, 2 mM L-Glutamine and 1% pen-strep at 37 °C in a 5% CO_2_ humidified environment. Culture medium was renewed every other day unless the 80% confluence level was reached. In this case, the cells were split using 0.25% trypsin-ethylenediaminetetraacetic acid (EDTA).

### 4.7. Cell Viability Assay

Evaluation of NP cytotoxicity was performed by CellTiter-Glo^®^ luminescent Cell Viability Assay (Promega, Belgium). For all in vitro studies, selected NPs (1:10 weight ratio of siRNA:Au for LBL-CS-AuNPs) were diluted in culture medium. Briefly, 100 µL of a suspension of 75,000 cells/mL were added in individual wells of 96-well flat-bottomed culture plates. After 24 h, different concentrations (2 nM–64 nM, effective siRNA concentration) diluted in complete RPMI-1640 (100 µL) were used to replace the culture medium and incubated for 4 h at 37 °C. Later, the cells were washed and incubated for another 24 h with fresh medium. Finally, the cell culture medium was removed and replaced with 100 µL of pre-heated CellTiter-Glo^®^ reagent and 100 µL of cell culture medium and shacked 10 min at 120 RPM at room temperature to induce complete cell lysis and to allow the signal to stabilize. After incubation, 100 µL suspension of each well was transferred to the white opaque 96 well plates and the luminescence signal was recorded by a GloMax™ 96 microplate Luminometer (Promega, Belgium). Data are presented as the mean cell viability (percentage of luminescent signal relative to non-treated cells (NTC) for each condition) ± standard deviation of the mean for minimum three independent repeats.

### 4.8. Apoptosis Assay by Flow Cytometry

Induction of apoptosis by the NPs was assessed using propidium iodide (PI) and dihexaoxacarbocyanine iodide (DiIC_1_(5)). In brief, cells were plated in 96-well plates at 7500 cells/well. The following day, the medium was replaced with different concentrations of NPs (2 nM–64 nM, effective siRNA concentration) which were diluted in fully supplemented RPMI-1640. Following 4 h incubation the cells were washed with PBS and incubated for another 24 h with fresh medium. After 24 h, the medium was removed and transferred to a U-bottom 96 well plate because the medium could contain dead cells, which should also be included in the analysis. After dissociating the remaining attached cells with trypsin solution, the removed cell medium is added again to neutralize trypsin activity and samples were centrifuged at 500 g for 5 min and the supernatant was decanted. Cell pellets were resuspended in staining buffer (Flow buffer supplemented with 1 µg/mL PI and 10 nM DiIC_1_(5)) and incubated for 30 min at 37 °C and analyzed by using the CytoFLEX flow cytometer with plate loader for 96-well plates (Beckman Coulter, Krefeld, Germany) and CytExpert software. The fluorescence intensity of DiIC_1_(5) and PI were measured at 638 nm and 482 nm excitation wavelength and 658 nm and 608 nm emission wavelength, respectively. The cell population was separated into three groups; live cells (high intensity of deep red fluorescence, DilC_1_(5)+/PI-); apoptotic cells (no fluorescence, DilC_1_(5)-/PI-); and dead cells (red fluorescence, DilC_1_(5)-/PI+) with FlowJo software (Tree Star Inc., Ashland, OR, USA).

### 4.9. Quantification of In Vitro Cellular NPs Internalization by Flow Cytometry

NP uptake by H1299 cells was evaluated using Alexa Fluor 647-labeled siRNA (si-AF647) loaded into NPs (as the second layer for CS-AuNPs). Cells were seeded in 96-well plates at 9000 cells/well and allowed to attach overnight. The next day, cells were treated with different concentrations of NPs containing si-AF647 (2 nM–64 nM, effective siRNA concentration) for 4 h. Next, cells were washed with PBS and detached from the well plates using trypsin/EDTA 0.25%, diluted with complete cell culture medium, transferred to U-Bottom 96-well plates, centrifuged at 500 g for 5 min and then cell pellets were resuspended in flow buffer (DPBS supplemented with 0.1% sodium azide and 1% BSA). Red fluorescence (638 nm excitation with argon laser and detection with a 660/20 nm bandpass filter) of samples was measured on a minimum of 10,000 cells using the CytoFLEX flow cytometer. For calculating rMFI, the following equation was used:

(1) $$\mathrm{rMFI}\;\left(\mathrm{relative}\;\mathrm{Mean}\;\mathrm{Fluorescence}\;\mathrm{Intensity}\right)=\frac{\mathrm{MFI}\;\mathrm{of}\;\mathrm{cells}\;\mathrm{treated}\;\mathrm{with}\;\mathrm{si}\_\mathrm{AF}647}{\mathrm{MFI}\;\mathrm{of}\;\mathrm{cells}\;\mathrm{treated}\;\mathrm{with}\;\mathrm{non}\_\mathrm{labeled}\;\mathrm{siRNA}}$$

### 4.10. Quantification of Transfection Efficiency by Flow Cytometry

H1299 cells stably expressing GFP (H1299-eGFP) were used to evaluate siRNA gene silencing efficiency of NPs. H1299-eGFP cells were seeded in 96-well plate at 7500 cells per well. 24 h after seeding, cells were transfected for 4 h with various concentrations of siRNA (for every si-eGFP condition, a si-CTRL sample was included to account for potential off-target effects) diluted in fully supplemented RPMI-1640. Next, the medium was removed and replaced with fresh culture medium. Finally, after 24 h, cells were washed with DPBS and dissociated by trypsin treatment. For neutralizing trypsin, fully supplemented RPMI-1640 was added and the suspensions were transferred to a 96-well U-bottomed plate and centrifuged at 500 g for 5 min. The supernatant was descanted and cells were resuspended in flow buffer for direct analysis using flow cytometry. For calculating siRNA gene silencing efficiency, GFP expression was quantified as the following equation:

(2) $$\mathrm{GFP}\;\mathrm{Expression}\;\left(\%\right)=\frac{\mathrm{MFI}\;\mathrm{si}\_\mathrm{eGFP}\;\left(\mathrm{Mean}\;\mathrm{fluorescence}\;\mathrm{intensity}\;\mathrm{of}\;\mathrm{cells}\;\mathrm{treated}\;\mathrm{with}\;\mathrm{anti}\;\mathrm{GFP}\;\mathrm{siRNA}\right)}{\mathrm{MFI}\;\mathrm{si}\_\mathrm{CTRL}\;\left(\mathrm{Mean}\;\mathrm{fluorescence}\;\mathrm{intensity}\;\mathrm{of}\;\mathrm{cells}\;\mathrm{treated}\;\mathrm{with}\;\mathrm{CTRL}\;\mathrm{siRNA}\right)}\times 100$$

where lower GFP expression means higher knockdown efficiency.

### 4.11. Visualizing eGFP Expression with Confocal Microscopy

H1299-eGFP cells were seeded at 75,000 cells/mL in 35 mm diameter CELLview glass bottom microscopy dishes (Greiner Bio-One, Vilvoorde, Belgium). On the next day, after removal of the fully supplemented RPMI-1640, the cells were treated with si-eGFP loaded NPs and compared to benchmark transfection agents (Lipofectamine and jetPEI^®^) as well as naked si-eGFP. The incubation time was always 4 h (37 °C, 5% CO_2_) for each condition after which cells were washed with DPBS. Cells were kept in fresh fully supplemented RPMI-1640 for an additional 24 h. Next, before confocal imaging, cell nuclei were stained with Hoechst 33,342 staining (1 mg/mL in H_2_O; 1000× diluted) in PBS during 15 min at 37 °C and washed 2 times with PBS. Then cells were fixed with 4% paraformaldehyde during 15 min at room temperature. After a double washing step with DPBS, finally one drop of Vectashield antifade mounting medium (Vector Laboratories, Burlingame, VT, USA) for preserving fluorescence was added to each sample. A Nikon A1R HD confocal (Nikon, Japan), equipped with a laser box (LU-N4 LASER UNIT 405/488/561/640, Nikon Benelux, Brussels Belgium), detectors (A1-DUG-2 GaAsP Multi Detector Unit, GaAsp PMT for 488 and 561 and Multi-Alkali PMT for 647 and 405 nm), and a 20× air objective lens (CFI plan Apo VC 20×, NA 0.75, WD 1000 µm) (Nikon, Japan). Images were recording using the NIS Elements software (Nikon, Japan). The 408 and 488 nm laser lines were used to excite the Hoechst labeled nuclei and the GFP protein, respectively.

### 4.12. Visualization and Quantification of Endosomal Escape

Visualization and quantification of endosomal escape were performed based on a dequenching assay first published by Rehman et al. [48] and further optimized by Vermeulen et al. [47]. To this end, red-labeled fluorescent oligonucleotides (AF647 ONs) were incorporated into the NPs instead of siRNA. Upon endosomal escape, the labeled ONs will spread toward the cytoplasm and finally accumulate in the nucleus. A red fluorescent nucleus is then a sign that at least one endosomal escape event happened in a particular cell.

H1299-eGFP cells were seeded in 35 mm CELLview glass bottom microscopy dishes (Greiner Bio-One, Vilvoorde, Belgium) at a density of 75,000 cells per mL. Next day, after removal of the complete medium, the cells were treated with NPs or commercial transfection agents (Lipofectamine and jetPEI^®^) containing AF647 ONs. Cells incubated with naked AF647 ONs without carrier were included as a control. All conditions were incubated for 4 h (37 °C, 5% CO_2_) and then washed with DPBS after which cells were kept in fresh fully supplemented medium for an additional 24 h. On the following day, cell nuclei were stained with Hoechst 33,342 (1 mg/mL in H_2_O; 1000× diluted) in DPBS during 15 min at 37 °C and washed 2 times with DPBS. Finally, fresh fully supplemented RPMI-1640 was added, and cells were kept at 37 °C in a humidified atmosphere containing 5% CO_2_ until confocal imaging. The 408 nm, 633 nm and 488 nm laser lines were applied to excite the Hoechst-labeled nuclei, the fluorescence resulting from AF647 ONs and the GFP protein, respectively. During data analysis with ImageJ (FIJI) software [60], nuclei were detected in the blue channel by thresholding (applying the same offset values for every image), and intensity analysis (mean gray value) of the nuclear fluorescence signal in the red channel was performed. From this, the percentage of cells with a AF647 ON-positive nucleus was determined, which is the percentage of cells in which at least one carrier was able to release its cargo molecules in the cytosol. Data are represented as the percentage of cells with AF647 ON positive nuclei as determined from at least 500 cells in a minimum of 20 images.

### 4.13. Statistical Analysis

All experiments were performed in triplicate. All the results are reported as mean ± standard deviation. Statistical comparisons were performed using one-way ANOVA to compare multiple conditions and student t-test for direct comparison of 2 conditions; a *p*-value < 0.05 was considered statistically significant.

## 5. Conclusions

LBL-CS-AuNPs were synthesized by direct reduction with chitosan, and were used as a core for the design of a siRNA delivery vehicle based on a facile layer-by-layer assembly method. The results demonstrated that the gold core substantially improves colloidal stability and siRNA protection, leading to markedly improved siRNA silencing compared to nanocarriers prepared from chitosan alone. All together, these results indicate that LBL-CS-AuNPs could become a very promising carrier to deliver siRNA for therapeutic applications.

## Figures and Tables

**Table 1:** The different weight ratio and corresponding number ratio of siRNA to CS-AuNPs.

Ratio siRNA:Au (w:w)	siRNA ^1^/CS-AuNPs ^2^
1:0.5	2240
1:1.0	1120
1:2.5	450
1:5.0	224
1:7.5	149
1:10	112
1:12.5	89

^1^ The number of siRNA has been calculated based on molecular weight. ^2^ The concentration of the AuNPs in the dispersions was estimated from the extinction of the dispersions (Figure S1A).

**Table 2:** Correlation between the effective siRNA concentration and the corresponding LBL-CS-AuNPs concentration.

siRNA Concentration	Au Concentration ^1^	LBL-CS-AuNPs/mL ^2^	siRNA/mL ^3^
2 nM	1.3 μM	1.0 × 10^10^	1.2 × 10^12^
4 nM	2.7 μM	2.1 × 10^10^	2.4 × 10^12^
8 nM	5.5 μM	4.3 × 10^10^	4.8 × 10^12^
16 nM	10.9 μM	8.5 × 10^10^	9.6 × 10^12^
24 nM	16.4 μM	1.3 × 10^11^	1.4 × 10^13^
32 nM	21.9 μM	1.7 × 10^11^	1.9 × 10^13^
50 nM	34.2 μM	2.7 × 10^11^	3.0 × 10^13^
64 nM	43.8 μM	3.4 × 10^11^	3.8 × 10^13^

^1^ Au concentration was determined by atomic absorption spectroscopy. ^2^ The concentration of AuNPs in the dispersions was estimated from their extinction spectra (Figure S1A). ^3^ The number of siRNA per mL was calculated based on Avogadro’s number.

## Data Availability

The data presented in this study are available on request from the corresponding author.

## Supplementary Materials

Supplementary Materials can be found at https://www.mdpi.com/1422-0067/22/2/831/s1.

## Abbreviations

AF647 ONs	Alexa Fluor 647-labeled oligonucleotides
CS	Chitosan polymer
CS-AuNP	Chitosan-coated gold nanoparticles
CSNPs	Chitosan nanoparticles
ddi. water	Distilled De-Ionized water
DiIC_1_(5)	Dihexaoxacarbocyanine iodide
DLS	Dynamic light scattering
DMEM	Dulbecco’s Modified Eagle Medium
DPBS	Dulbecco’s Phosphate-Buffered Saline
EDTA	Ethylenediaminetetraacetic acid
eGFP	*Enhanced green fluorescent protein*
FBS	*Fetal bovine serum*
FTIR	Fourier-transform infrared spectroscopy
LBL	Layer by layer
LBL-CS-AuNP	Layer by Layer chitosan-coated gold nanoparticles
LSPR	Localized surface plasmon resonance
NTC	Not treated control
PEI	Polyethylenimine
PI	Propidium iodide
RISC	RNA-induced silencing complex
rMFI	Relative mean fluorescence intensity
RPMI	Roswell Park Memorial Institute
SDS	Sodium dodecyl sulfate
si-AF647	Alexa Fluor 647-labeled siRNA
siRNA	Small interfering RNA
TEM	Transmission electron microscopy
TPP	Tripolyphosphate
UV-Vis	Ultraviolet–visible spectroscopy
