# Supplementary material for: Layer by Layer Assembled Chitosan-Coated Gold Nanoparticles for Enhanced siRNA Delivery and Silencing
Source: Int J Mol Sci. 2021 Jan 15;22(2):831. doi: 10.3390/ijms22020831 (PMC7830320; doi:10.3390/ijms22020831)
Supplement: Supplementary file 1 [file ijms-22-00831-s001.pdf]

Supplementary Materials

Layer by Layer Assembled Chitosan-Coated Gold Nanoparticles for Enhanced siRNA Delivery and Silencing

Elnaz Shaabani [1,2], Maryam Sharifiaghdam [1,2], Herlinde De Keersmaecker [2,3], Riet De Rycke [4,5], Stefaan De Smedt [2,3], Reza Faridi-Majidi [1,*], Kevin Braeckmans [2,3,*] and Juan C. Fraire [2]

1 Department of Medical Nanotechnology, School of Advanced Technologies in Medicine, Tehran University of Medical Sciences, Tehran, Iran; elnaz.shaabanisichani@ugent.be (E.Sh.); maryam.sharifiaghdam@ugent.be (M.Sh.)
2 Laboratory of General Biochemistry and Physical Pharmacy, Faculty of Pharmacy, Ghent University, B-9000 Ghent, Belgium; herlinde.dekeersmaecker@ugent.be (H.D.K.); stefaan.desmedt@ugent.be (S.D.S.); juan.fraire@ugent.be (J.C.F.)
3 Center for Advanced Light Microscopy, Ghent University, 9000 Ghent, Belgium
4 Department of Biomedical Molecular Biology, Ghent University, 9052 Ghent, Belgium and VIB Center for Inflammation Research, 9052 Ghent, Belgium; riet.derycke@ugent.be
5 Ghent University Expertise Centre for Transmission Electron Microscopy and VIB BioImaging Core, 9052 Ghent, Belgium
* Correspondence: refaridi@sina.tums.ac.ir (R.F.-M.); Kevin.Braeckmans@UGent.be (K.B.)

The AuNPs were characterized by UV-vis spectroscopy, dynamic light scattering (DLS), TEM and electrodynamic modeling using Mie theory.

The optical extinction of AuNPs was computed using Mie theory. In particular, we used the generalized multiparticle Mie theory (GMM) formulation developed by Xu [1]. The calculation was performed using the dielectric function tabulated by Palik for Au, [2] the size determined by TEM (15 nm), and considering that particles were immersed in water (refractive index n_r = 1.33).

The concentration of the AuNPs in the dispersion was estimated from the experimental extinction as measured at the maximal extinction wavelength (Ext), as follows (Equation 1) [3]:

$$[AuNPs]\left(\frac{number}{mL}\right) = \frac{Extinction}{b \times \sigma EXT}$$

Where "b" is the optical path (1 cm) and "σEXT" (cm^2) is the theoretical extinction cross section for spherical particles obtained by Mie theory calculations [4].

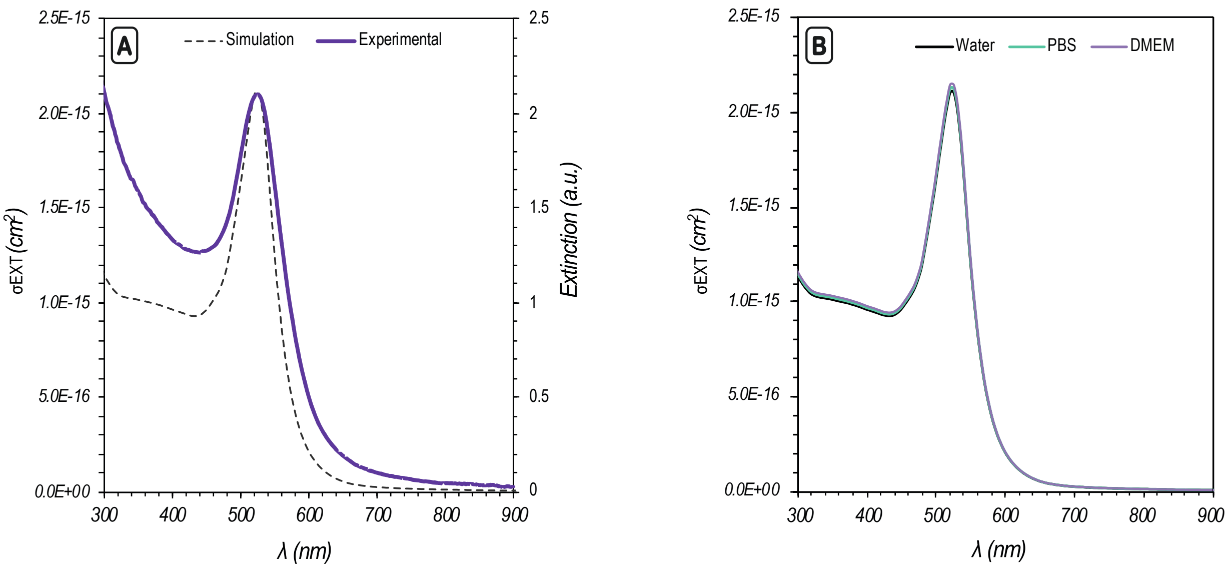

Figure. S1: (A) Experimental extinction spectrum of synthesized CS-AuNPs and simulated extinction of spherical 15 nm AuNPs. (B) Mie theory simulations of the extinction cross-section spectrum of 15 nm AuNPs in different media: water (refractive index = 1.33); PBS (refractive index = 1.3348); DMEM (refractive index = 1.3370).

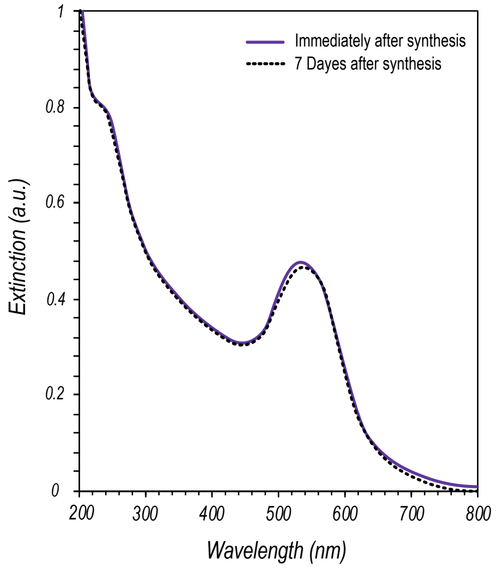

Figure. S2: UV-Vis spectrum of freshly synthesized CS-AuNPs and 7 days after synthesis.

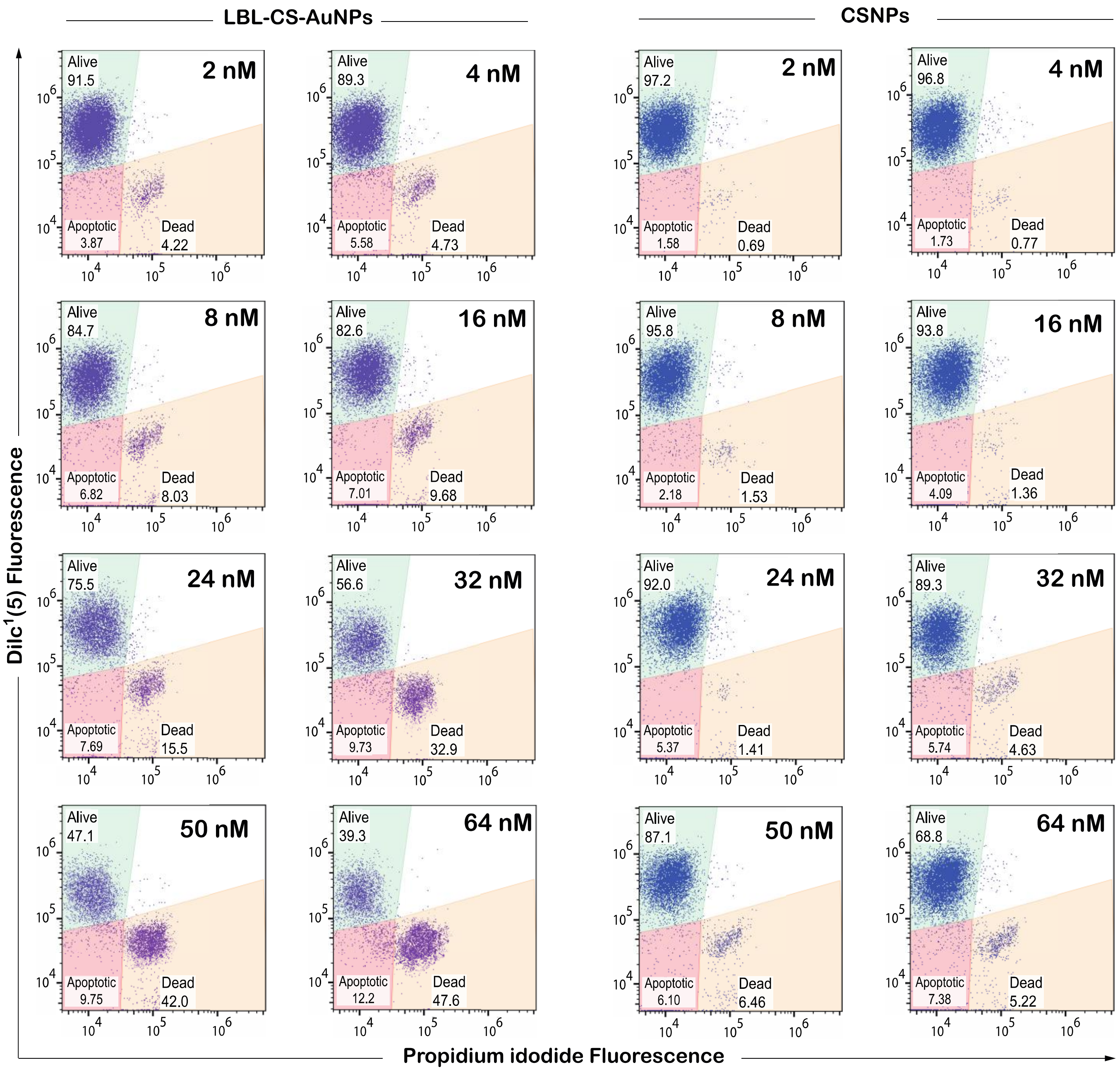

Figure. S3: Comparison of apoptosis between LBL-CS-AuNPs and CSNPs. Flow cytometric dot plots of apoptosis events in H1299-eGFP cells after treatment with NPs at the indicated effective siRNA concentrations.

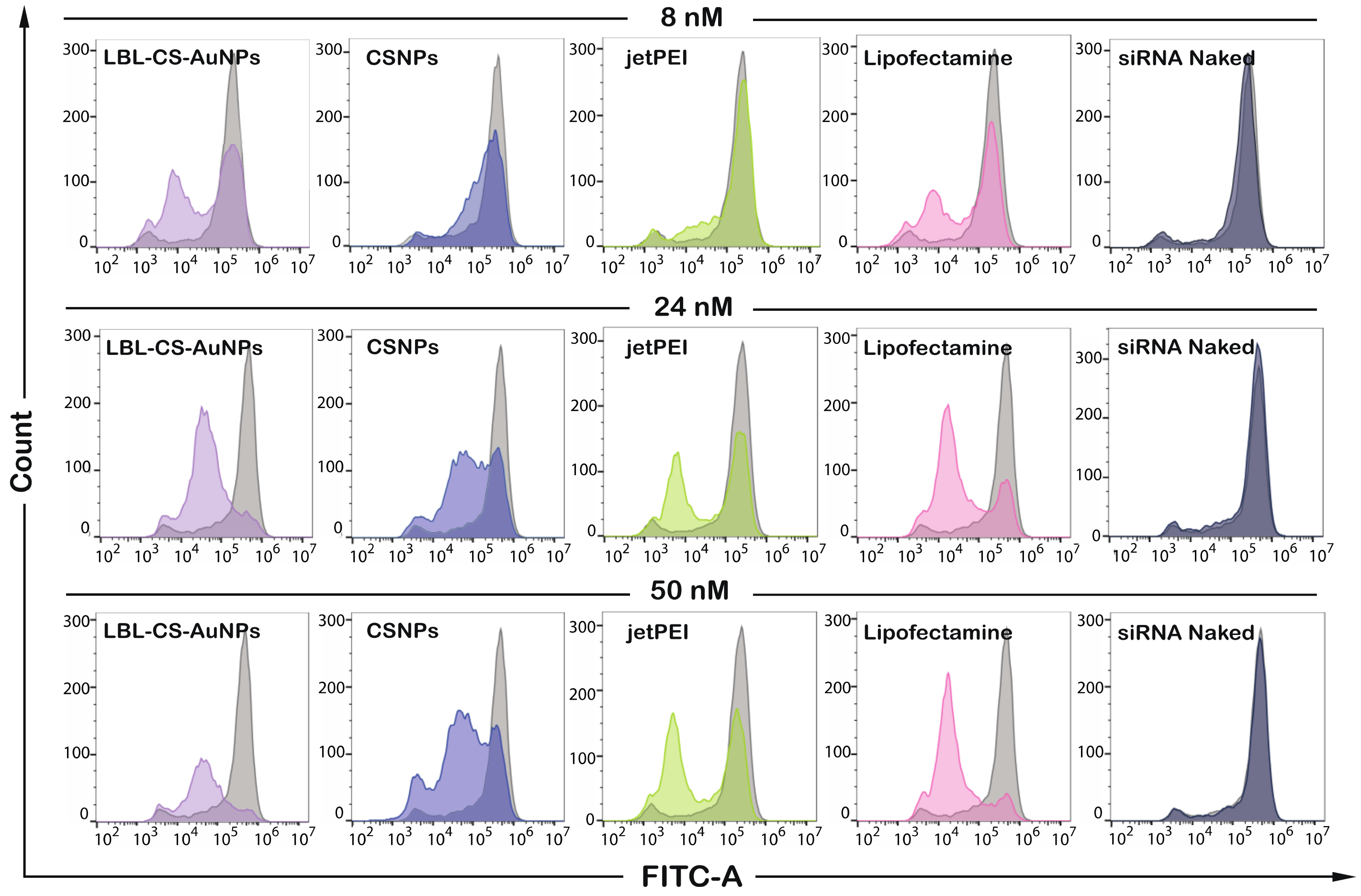

Figure. S4: Evaluating the knockdown efficacy of LBL-CS-AuNPs and CSNPs in comparison with jetPEI and Lipofectamine as commercial transfection reagents for siRNA. (C) Flow cytometry histograms of cells treated with different carriers at 8 nM, 24 nM, and 50 nM concentration of siRNA.

References:

1. Xu, Y.-l.; Wang, R.T. Electromagnetic scattering by an aggregate of spheres: Theoretical and experimental study of the amplitude scattering matrix. *Physical Review E* **1998**, *58*, 3931.
2. Palik, E.D. *Handbook of optical constants of solids*; Academic press: 1998; Vol. 3.
3. Haiss, W. thanh nt K., Aveyard J., Fernig DG. *Analytical chemistry* **2007**, *79*, 4215-4221.
4. Xu, Y.-l.; Gustafson, B.Å. A generalized multiparticle Mie-solution: further experimental verification. *Journal of Quantitative Spectroscopy and Radiative Transfer* **2001**, *70*, 395-419.
